# Development of an Immunochromatographic Test Based on Rhoptry Protein 14 for Serological Detection of *Toxoplasma gondii* Infection in Swine

**DOI:** 10.3390/ani12151929

**Published:** 2022-07-28

**Authors:** Yimin Yang, Yechuan Huang, Xianfeng Zhao, Mi Lin, Lulu Chen, Mingxiu Zhao, Xueqiu Chen, Yi Yang, Guangxu Ma, Chaoqun Yao, Siyang Huang, Aifang Du

**Affiliations:** 1 Zhejiang Provincial Key Laboratory of Preventive Veterinary Medicine, Institute of Preventive Veterinary Medicine, College of Animal Sciences, Zhejiang University, Hangzhou 310058, China; yiminyang0412@zju.edu.cn (Y.Y.); yechuanhuang@163.com (Y.H.); linmi@sibcb.ac.cn (M.L.); luluchen@zju.edu.cn (L.C.); 11817029@zju.edu.cn (M.Z.); chenxueqiu@zju.edu.cn (X.C.); yangyi0607@zju.edu.cn (Y.Y.); gxma1@zju.edu.cn (G.M.); 2 Animals & Plant Inspection and Quarantine Technology Center of Shenzhen Customs, Shenzhen 518045, China; zxf0903@126.com; 3 School of Veterinary Medicine and One Health Center for Zoonoses and Tropical Veterinary Medicine, Ross University School of Veterinary Medicine, Basseterre P.O. Box 334, Saint Kitts and Nevis; chyao@rossvet.edu.kn; 4 Jiangsu Co-innovation Center for Prevention and Control of Important Animal Infectious Diseases and Zoonosis, Jiangsu Key Laboratory of Zoonosis, Institute of Comparative Medicine, College of Veterinary Medicine, Yangzhou University, Yangzhou 225009, China; siyang.huang@hotmail.com

**Keywords:** *Toxoplasma gondii*, immunochromatographic test, TgROP14, swine

## Abstract

**Simple Summary:**

Toxoplasmosis is one of the most common parasitic zoonoses in the world. It not only threatens human health and safety but also causes considerable losses to the global animal husbandry industry. Swine, as an intermediate host of *Toxoplasma gondii*, has important economic and public health significance, and the global prevalence of *T. gondii* in swine varies between 10% and 60%. Therefore, it is important to establish simple, effective, sensitive and specific diagnostic methods for the detection and prevention of *T. gondii* infection in swine. In the present study, we developed a colloidal gold immunochromatographic strip based on rTgROP14, recombinant protein A and monoclonal antibody TgROP14-5D5 for serological detection of *T. gondii* in swine populations. This new ICT achieved good specificity and sensitivity and has potential for routine serological detection of *T. gondii* in swine.

**Abstract:**

*Toxoplasma gondii*, a worldwide distributed apicomplexan protozoan, can infect almost all warm-blooded animals and may cause toxoplasmosis. In order to provide a point-of-care detection method for *T. gondii* infection, an immunochromatographic test (ICT) was established. The proposed test uses recombinant *T. gondii* rhoptry protein 14 (ROP14) conjugated with 20 nm gold particles, recombinant protein A as the detection line and monoclonal antibody TgROP14-5D5 as the control line. The specificity, sensitivity, positive predictive value, negative predictive value and stability of this new ICT were evaluated. rTgROP14 was specifically recognized by positive serum of *T. gondii* but not negative serum. mAb TgROP14-5D5 showed higher specific recognition of *T. gondii* antigens and was therefore selected for subsequent colloidal gold strip construction. The new ICT based on TgROP14 exhibited good diagnostic performance with high specificity (86.9%) and sensitivity (90.9%) using IHA as a “reference standard”. Among 436 field porcine sera, ICT and IHA detected 134 (30.7%) and 99 (22.7%) positive samples, respectively. The relative agreement was 87.8%. These data indicate that this new ICT based on TgROP14 is a suitable candidate for routine testing of *T. gondii* in the field.

## 1. Introduction

*Toxoplasma gondii* is a worldwide distributed apicomplexan, which can parasitize nearly all types of warm-blooded animals, including humans [1]. Approximately one-third of the human population globally has been infected by *T. gondii* [2]. *T. gondii* infection in healthy adults is usually asymptomatic; however, devastating consequences often occur in the congenital infections and in people with compromised immunity [3]. The majority of human *T. gondii* infections are caused by oral ingestion of raw or inadequately cooked meat containing viable tissue cysts or as a result of consuming food or water contaminated with cat-shed oocysts [4,5]. Pigs (*Sus scrofa*), as an intermediate host of *T. gondii*, have important economic and public health significance [6,7], and tissue cysts can survive for more than two years, whereas most pigs are sacrificed as pork within a year [5]. Globally, the infection rate of *T. gondii* in pigs varies widely, typically between 10% and 60% [8]. China has one of the largest pork industries worldwide, with the prevalence of *T. gondii* infection in swine ranging from 24.5% in central China [9] to 58.1% in southern China [10]. Therefore, detection and prevention of swine toxoplasmosis is of considerable significance for agriculture and public health in China, as well as the rest of the world.

Diagnosis of *T. gondii* infection generally relies on laboratory testing of parasites, antibodies and/or DNA [11] using parasitological, immunological and molecular detection [12]. Immunological diagnostics are the preferred and most commonly used method of detection [13]. They include indirect hemagglutination test (IHA), latex agglutination test (LAT), enzyme-linked immunosorbent assays (ELISA) and immunofluorescence antibody test (IFAT) [14]. These methods are commonly used, despite certain limitations, such as the requirement for a skillful operator, expensive equipment and time-consuming processes [13] compared to point-of-care testing. The colloidal gold immunochromatographic test (ICT) is considered suitable for rapid indoor detection, as it is simple to operate, with no need for equipment, in addition to its small size and ease of storage [15]. Several ICTs have been developed for detection of *T. gondii* infections, including those based on surface antigen 2 (SAG2) [16], surface antigen 3 (SAG3) [17] or dense granule antigen protein 7 (GRA7) [18,19]. Different diagnostic antigens have their own limitations, as the expression patterns of antigen genes vary during different infection stages and among different *T. gondii* strains [20,21]. More diagnostic antigens and methods should be identified and evaluated to maximize detection of all serologically positive individuals in the future.

Rhoptry proteins of *T. gondii* (TgROPs), such as ROP1 and ROP2, are potential diagnostic antigens [22,23,24,25]. Among them, TgROP14 is located in the parasitophorous vacuole membrane (PVM), possibly serving as a membrane transporter participating in the exchange of substances between the parasitophorous vacuole (PV) and the host cell cytoplasm [26]. We aimed to use TgROP14 as a key molecule in a reliable yet quick and user-friendly diagnostic method for detection of *T. gondii* infection that can be used at point of care.

## 2. Methods

### 2.1. Ethical Statement

Approval for the use of mice was received from Zhejiang University Experimental Animal Ethics Committee (Permit Number: ZJU201308-1-10-072). Mouse research was carried out in accordance with the recommendations presented in the Regulations for the Administration of Affairs Concerning Experimental Animals of the People’s Republic of China.

### 2.2. Parasites and Cell Culture

African green monkey kidney epithelial (Vero) cells (ATCC, CCL-81™) were maintained in Dulbecco’s Modified Eagle Medium (DMEM, Bioind, Israel) supplemented with 10% (*v*/*v*) fetal bovine serum (FBS, Gibco, Carlsbad, CA, USA). Tachyzoites of RHΔ*ku80*Δ*hxgprt* strain were cultured *in vitro* by serial passage on Vero cells at 37 °C and 5% CO_2_, with DMEM supplemented with 2% (*v*/*v*) FBS.

### 2.3. Soluble Protein Preparation

Freshly harvested *T. gondii* tachyzoites were washed thrice in phosphate-buffered saline (PBS), sonicated 5 × 20 s at 5 kHz and centrifuged at 12,000∗ *g* for 10 min to collect the supernatant containing *T. gondii* soluble antigens.

### 2.4. Western Blot

Approximately 10 μg *T. gondii* soluble protein or 1 μg recombinant TgROP14 protein was loaded into each lane by sodium dodecyl sulfate-polyacrylamide gel electrophoresis (SDS-PAGE) with 12% gel and transferred to 0.22 μm PVDF membranes (Millipore, Darmstadt, Germany). Membranes were blocked with 5% (*w*/*v*) skim milk for 1 h at room temperature and probed with mouse anti-TgROP14 antibodies (1:2000) (prepared in this study), mouse anti-His antibody (1:1000) (Beyotime, Shanghai, China) or mouse anti-*T. gondii* antibodies (1:1000) (conserved in our laboratory), followed by horseradish peroxidase (HRP)-conjugated goat anti-mouse IgG (1:5000) (Fudebio, Hangzhou, China) for 1 h. Blots were exposed using ECL substrates (Fudebio, Hangzhou, China) and visualized using the ChemiDoc™ chemiluminescence system (Bio-Rad, Hercules, CA, USA).

### 2.5. Preparation of the Recombinant Protein TgROP14 (rTgROP14-His)

According to the manufacturer’s instructions, total RNA of *T. gondii* RHΔku80Δhxgprt tachyzoites was extracted with TRIzol (Invitrogen, Carlsbad, CA, USA) and reverse-transcribed into cDNA using a ReverTra Ace-α-^®^ reverse transcription (Toyobo Co Ltd., Osaka, Japan). The transmembrane domains of TgROP14 protein were predicted by TMHMM Server v.2.0 (http://www.cbs.dtu.dk/services/TMHMM/, access at 12 October 2019). An insert corresponding to amino acids 619-1061 of TgROP14 (GenBank accession number DQ096565.1) without the transmembrane domains was amplified by PCR, using cDNA as template with following primers: 5′-CCGGAATTCATGCCAGACCAGGTTATGGATTCAG-3′ and 5′-CCCAAGCTTCAGCGCTTGCTTCTTCCTAGTC-3′, including an EcoR I and a Hind III restriction enzyme site underlined. EcoR I- and Hind III-digested PCR products were cloned into the *Escherichia coli* expression vector pET-30a (Novagen, Beijing, China). The right construct confirmed by sequencing was transformed into *E. coli* BL21 (DE3) (Takara Bio Inc., Otsu, Shiga, Japan) to express rTgROP14-His by 1 mM IPTG induction at 37 °C. After bacterial sonication in PBS (0.1 M, pH 7.4), rTgROP14-His was purified by Ni-NTA agarose (GE Healthcare Life Sciences, Marlborough, MA, USA). The quality of rTgROP14-His was verified by SDS-PAGE and WB.

### 2.6. Production of Mouse Monoclonal Antibodies against TgROP14

For mAb preparation, six-week-old female BalB/c mice (Shanghai SLAC Laboratory Animals Co., Ltd., Shanghai, China) were immunized with purified rTgROP14-His; then, the immunized spleen cells of mice were isolated and fused with SP2/0 myeloma cells [27]. Briefly, for the first immunization, each mouse received 100 μg of purified rTgROP14-His mix with an equal volume of complete Freund’s adjuvant. For the second and third immunizations, the mice were immunized with the same dosage of purified rTgROP14-His mixed in a 1:1 ratio with incomplete Freund’s adjuvant, both at 2-week intervals. One week after the third immunization, a booster intraperitoneal injection was administered with only 50 μg rTgROP14-His. Three days later, harvested spleen cells were fused with SP2/0 myeloma cells by polyethylene glycol.

Hybridomas were screened by ELISA to produce monoclonal antibodies (mAbs) against TgROP14 using rTgROP14-His to coat the wells. ELISA-positive hybridomas were further screened by IFAT. Briefly, *T. gondii* tachyzoite-infected Vero cells were fixed in 4% paraformaldehyde (PFA) for 15 min at 4 °C, permeabilized with PBS containing 0.25% Triton X-100 for 15 min at 37 °C and blocked in 1% bovine serum albumin (BSA) for 1 h at 37 °C. Subsequently, they were incubated both for 1 h in mAbs anti-TgROP14 (1:1000) and Alexa Fluor 488-conjugated secondary antibodies (1:1000) and stained with DAPI (4′,6-diamidino-2-phenylindole). Finally, fluorescence images were obtained by a laser scanning confocal microscope (Zeiss LSM 780, Jena, Germany). Identified positive hybridomas were subcloned three times by limiting dilution method. Ascites were generated in the paraffin-primed BalB/C mice and purified using saturated ammonium sulfate [(NH_4_)_2_SO_4_]. The quality and specificity of the mAbs were tested by SDS-PAGE and WB.

### 2.7. Identification of Types and Epitope Specificities of the mAbs

A mouse monoclonal antibody isotyping kit (Biodragon, Beijing, China) was selected to determine the isotypes of the mAbs. The epitope specificities of the two prepared mAbs were tested by an ELISA overlap experiment, and additivity indices (A.I.s) of different combinations of mAbs were calculated using previously described methods [28].

### 2.8. Preparation of Immunoassay Materials

First, colloidal gold solution was prepared. Briefly, 2.5 mL of 1% trisodium citrate solution was supplemented into 100 mL of boiled 0.01% HAuCl_4_ solution by stirring thoroughly and continuously. The mixture was boiled for an additional 10 min after its color turned from blue to dark red. Then, the mixture was constantly stirred for another 5 min before the preparation was completed. The prepared gold colloids were stored in the dark at 4 °C with 0.01% (*m*/*v*) sodium azide (NaN_3_). Transmission electron microscopy was used to identify the gold colloids.

The desalted recombinant protein rTgROP14-His was used as an antibody detector after being conjugated with the colloidal gold, whereas staphylococcal protein A (Sangon Biotech, Shanghai, China) was used as the capture protein. The optimum conditions were determined as follows: 0.2 mL purified and desalted rTgROP14-His (1.5 mg/mL) was supplemented into 20 mL of colloidal gold solution (pH 8.2). The mixture was stirred carefully for 15 min and blocked by 10% BSA (*m*/*v*) for 1 h. After centrifugation (12,000∗ *g*, 30 min), the colloidal gold–antigen conjugate from the sediment was resuspended in 2 mL dilution buffer (0.2 M tris solution, pH 8.0, with 10% BSA, 20% sucrose, 5% trehalose and 0.2% NaN_3_) and stored at 4 °C. The recombinant protein A (4 mg/mL) and TgROP14-5D5 mAb (2 mg/mL) were transferred to a nitrocellulose (NC) membrane (Millipore, Darmstadt, Germany) at a rate of 1 μL/cm, forming the test and control lines, respectively. The strips were incubated and dried at 37 °C for 1 h.

### 2.9. Preparation of the Immunochromatographic Strip

**Figure 1:**
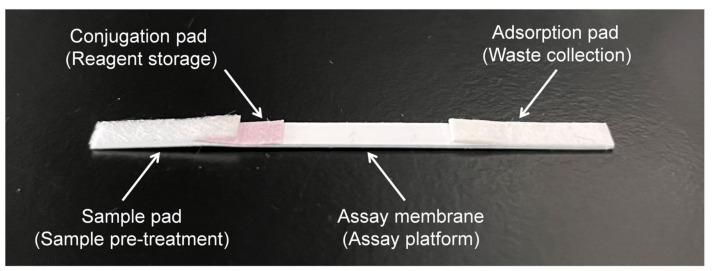
The component and illustration of the ICT strip.

The ICT strip was assembled as showed in Figure 1. It consisted of a sample pad saturated with 0.01 M PBS (pH 8.2) containing 0.1% Tween-20, a conjugate pad combined with a colloidal gold probe, an immobilized NC membrane and an absorbent pad. Both the sample pad and the conjugate pad, along with the NC membrane prepared as described above, were dried at 37 °C. Pure cellulose fiber served as the absorbent pad, and the PVC plate was used as the assay membrane at the bottom of the test strip. These strips were sequentially overlapped with the sample pad, conjugate pad, fixed NC membrane and absorbent pad; cut into 4 mm widths; and stored with desiccators at 4 °C until use.

A sample in 80 μL was dropped onto the sample pad. It passed through the NC membrane and, a result, showed up in 10 min. Both the test and control zones showing red lines indicated a positive result, whereas only the control zone appearing as red denoted a negative result. A single red line in the test zone or no red line at all on the strip indicated an invalid test.

### 2.10. Sensitivity, Specificity and Stability of the Immunochromatographic Test

To identify the detection limit of ICT, the standard *T. gondii*-positive porcine sera were diluted in a ratio of 1:2, 1:4 and 1:8 with 0.9% NaCl (pH 7.2) and the standard negative porcine serum as the negative control. The specificity of ICT was evaluated using positive porcine sera for *Isospora suis* (Is), *Cryptosporidium suis* (Cs), *Neospora caninum* (Nc), porcine reproductive and respiratory syndrome virus (PRRSV), swine type-O foot-and-mouth disease virus (FMDV O-type), swine type-A foot-and-mouth disease virus (FMDV A-type) and swine fever virus (SFV), and pig anti-*T. gondii*-positive and -negative sera were set as controls. The positive porcine sera for parasites were kept in our laboratory, and the positive porcine sera for viruses were kindly provided by Zhejiang Animal Disease Prevention and Control Center, China. The strips stored at 4 °C for 12 weeks were used to examine the stability of this ICT.

### 2.11. Detection of T. gondii Infection in Field Samples

A total of 436 porcine sera sampled from Zhejiang Province in China were tested using the new ICT. They were also detected by an IHA kit (Lanzhou Veterinary Research Institute, Chinese Academy of Agricultural Sciences, Lanzhou, Gansu Province, China), serving as a “reference standard” to assess the relative sensitivity and specificity of the newly developed ICT. This commercial IHA kit is a key item of China national standard GB/T 18448.2-2008 for the detection of antibodies to *T. gondii* in animals and obtained ISO9001:2015 international certification; it has been used to detect *T. gondii* antibodies in many animals [29,30]. The relative sensitivity of the ICT was calculated by the percentage of positive samples with consistent results relative to the number of positive samples with the IHA kit. The relative specificity of the ICT was calculated by the percentage of negative samples with consistent results relative to the number of negative samples with the IHA kit. The positive predictive value (PPV) of the ICT was calculated by the percentage of positive samples with consistent results relative to the number of positive samples with the ICT. The negative predictive value (NPV) of the ICT was calculated by the percentage of negative samples with consistent results relative to the number of negative samples with the ICT. Furthermore, the composite reference standard was also used to evaluate the new ICT. Samples that were detected as positive by either ICT or IHA were considered composite reference-standard-positive, and samples detected as negative by both ICT and IHA were considered composite reference-standard-negative. Sensitivity, specificity, PPV and negative predictive value (NPV) were calculated accordingly. Moreover, the agreement between the tests was calculated by the percentage of samples with consistent results detected by the two methods relative to the total number of samples.

## 3. Results

### 3.1. rTgROP14-His Proteins Are Recognized by Antibodies to T. gondii

**Figure 2:**
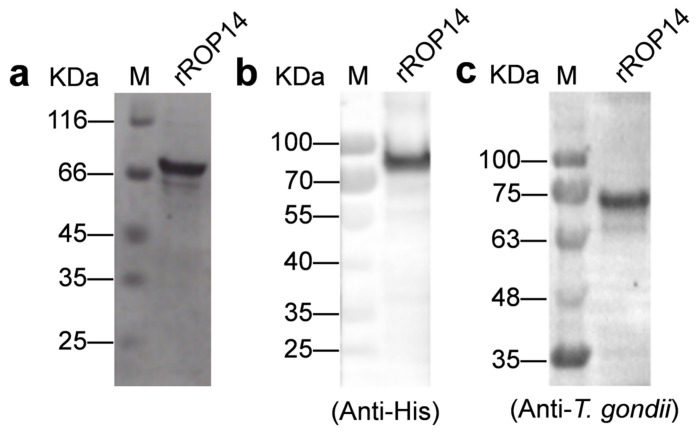
Analysis of the recombinant protein rTgROP14-His by SDS-PAGE (**a**) and Western blot (**b**,**c**). M: protein marker, rROP14: rTgROP14-His. The primary antibodies in panels b and c are anti-His antibody and polyclonal mouse antibodies to *T. gondii*, respectively.

The amino acids 619-1061 of ROP14 were chosen for preparation of rTgROP14-His, as they do not include the transmembrane domains of TgROP14 predicted by TMHMM Server v.2.0 (Figure S1). The recombinant protein rTgROP14-His was generated by transfecting *E. coli* BL21 (DE3) with the plasmid pET30a-TgROP14-His. It was approximately 70 kDa, as confirmed by SDS-PAGE (Figure 2a) and Western blot with anti-His antibody (Figure 2b and Figure S2). Furthermore, rTgROP14-His was recognized by mouse polyclonal antibodies to *T. gondii* (Figure 2c and Figure S2), which indicated that rTgROP14-His has good immunogenicity.

### 3.2. TgROP14-5D5 Is a Candidate for Colloidal Gold Strip

**Figure 3:**
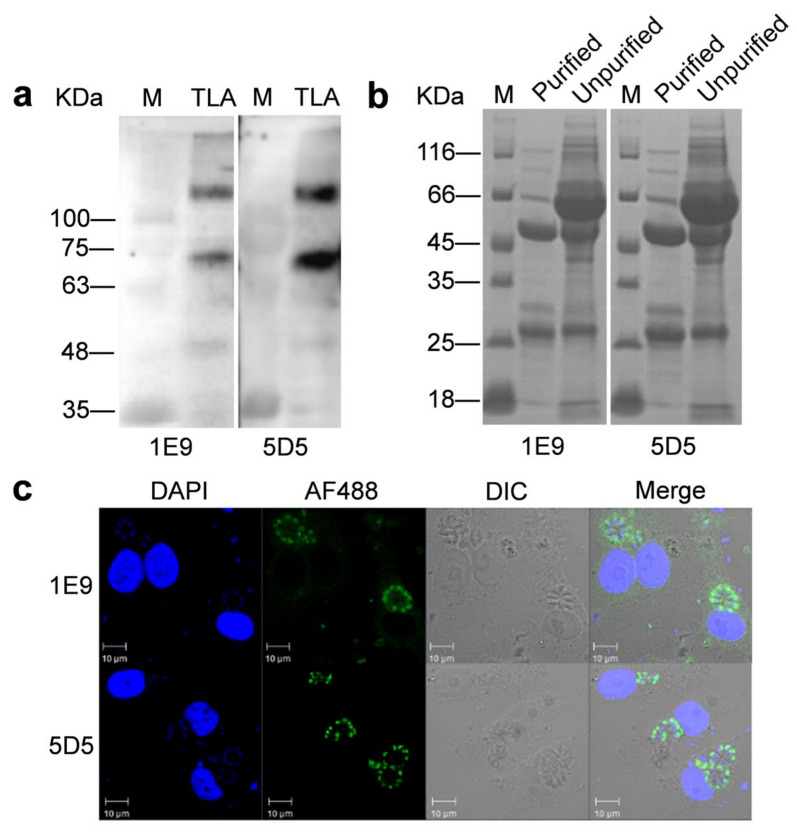
Identification of TgROP14-5D5 mAb and TgROP14-1E9 mAb. (**a**) Western blot analysis of two mAbs reacted with *T. gondii* lysate antigen (TLA). Total soluble proteins of *T. gondii* tachyzoites (10 μg) were loaded into each lane. The primary antibodies were mAb 1E9 and 5D5 to TgROP14. M: protein marker. (**b**) SDS-PAGE analysis of two mAbs before and after purification. Left: 1E9 mAb after purification and before purification; right: 5D5 mAb after purification and before purification; M: protein marker. (**c**) IFAT of two mAbs. Vero cells were used after infection with *T. gondii* tachyzoites for 24 h. The primary antibodies and Alexa Fluor 488-conjugated secondary antibodies for labeling were used in 1:1000 dilution. Confocal micrographs are shown.

Two mAbs, 1E9 and 5D5, against rTgROP14-His were characterized using the natural antigens of *T. gondii* tachyzoites by Western blot. Both detected two proteins at approximately 140 and 70 kDa (Figure 3a and Figure S3), and it is possible that the upper band is the dimer of the lower band; alternatively, they were the same proteins with different post-translational modifications. They were further determined to be IgG3 and IgG2a, respectively (Table 1). The two mAbs were successfully purified by saturated ammonium sulfate, as shown by the heavy chain (~55 kDa) and the light chain (~25 kDa) in SDS-PAGE (Figure 3b). Furthermore, the value of A.I was 22.8%, which demonstrated that these two mAbs targeted the same epitope (Table 1). Specificities of these two mAbs were further analyzed by IFAT, and TgROP14-5D5 showed higher specific recognition of *T. gondii* antigens than TgROP14-1E9 (Figure 3c). TgROP14-5D5 had a titer of approximately 1:3.3 × 10^7^ and was selected for subsequent colloidal gold strip construction because of its higher specificity.

### 3.3. Immunochromatographic Test Using TgROP14-5D5 Is Sensitive and Specific

**Figure 4:**
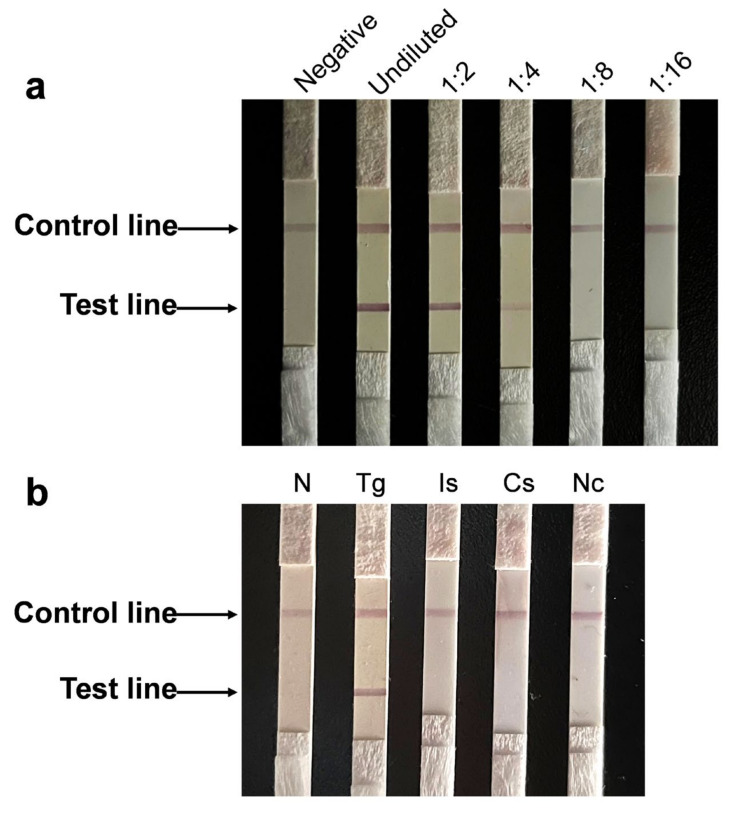
Sensitivity and specificity of the ICT strip. (**a**) Sensitivity of the developed ICT. From left to right: standard *T. gondii*-negative porcine serum, standard undiluted *T. gondii*-positive porcine serum; 1:2, 1:4, 1:8 and 1:16 dilution, respectively. (**b**) Specificity of the developed ICT using sera of pigs infected with different pathogens as specified. From left to right: negative control, *T. gondii* (Tg), *I. suis* (Is), *C. suis* (Cs) and *N. caninum* (Nc).

The sensitivity of the ICT was gauged by a series of 1:2 diluted *T. gondii*-positive porcine serum samples up to 1:16. Red color at the testing line was clearly observed when the sera were diluted to 1:4 (Figure 4a), indicating that the detection limit of the ICT was 1:4. The specificity of the ICT was verified with porcine serum samples positive for different pathogens that were often clinically observed. They included *I. suis*, *C. suis*, *N. caninum*, PRRSV, FMDV O-type, FMDV A-type and SFV. Two red lines were only observed with the *T. gondii*-positive porcine serum in the test and control zones. In contrast, only the control line appeared when each of the other sera were tested (Figure 4b and Figure S4). Furthermore, a red line was clearly observed in the test band exposed to positive porcine serum with the same batch of ICT strips stored at 4 °C for 12 weeks (data not shown), indicating that the test strips stored at 4 °C were stable for at least 12 weeks.

### 3.4. Newly Developed Immunochromatographic Test Is Suitable for Field Samples

We next compared the newly developed ICT test with a commercially available IHA as a “gold standard”. We used 436 porcine sera collected from farm pigs in Zhejiang, China. The number of positive and negative samples detected by the ICT and IHA methods were compared and are shown in Table 2. The new ICT identified 134 positive sera (30.7%). In contrast, IHA detected 99 positive sera, representing 22.7% of all samples (Table 3). The relative sensitivity and specificity of this newly prepared ICT were 90.9% and 86.9%, respectively, using the IHA alone as the reference standard (Table 4). When evaluated using the composite reference standard, the relative sensitivity and specificity of this new ICT were 93.7% and 100%, respectively (Table 4). PPV and NPV were also calculated using the composite reference standard and using the ICT alone as the reference standard (Table 4). The number of samples with consistent results detected by the two methods was 383, and the relative agreement between ICT and IHA was 87.8%.

## 4. Discussion

Over the past 30 years, many recombinant antigens of *T. gondii* as diagnostic targets have been assessed to detect specific antibodies in human serum [11,31]. Some researchers also evaluated the feasibility of utilizing recombinant *T. gondii* antigens for the detection of specific antibodies in animal sera [32,33]. ROPs of *T. gondii*, such as ROP1 and ROP2, have been used as detection antigens for diagnosis [22,23,24,25]. The recently discovered ROP14 may be a membrane transporter and is likely located in the PVM and participates in substance exchange between *T. gondii* and host cells [26]. In this study, the DNA sequence encoding TgROP14 minus transmembrane domain was PCR-amplified and cloned. The resultant recombinant protein rTgROP14-His was obtained by the *E. coli* prokaryotic expression system. Western blot analysis showed that the rTgROP14-His without a transmembrane domain could be specifically recognized by the positive serum of *T. gondii*, indicating that rTgROP14-His is a potential candidate for a diagnostic antigen. Using rTgROP14-His as the immunogen, two mAbs that can recognize the natural antigen of *T. gondii* were screened. The A.I. value of TgROP14-5D5 and TgROP14-1E9 was 22.8%, demonstrating that these two mAbs target the same epitope and that they are not suitable for establishing double-antibody sandwich immunological detection methods. Furthermore, based on TgROP14-5D5 showing higher specific recognition than TgROP14-1E9 by IFAT, it was chosen for ICT construction.

Several serologic kits are commercially available for the diagnosis of *T. gondii* infection, including LAT, modified agglutination test (MAT), Western blotting test and IFAT. Whereas several new laboratory diagnostic methods have been established for the serological detection of *T. gondii* in animals, they are not yet used clinically at a large scale. A novel luciferase-linked antibody capture assay (LACA) [34] and a fluorescent bead-based multiplex assay [35] were recently established for the individually serological diagnosis of *T. gondii* in pigs and chickens. In addition, a miniaturized protein microarray assay was recently developed for analysis of antibodies in meat juice and blood serum with high sample throughput for the slaughter of pigs [36]. At present, most serologic methods require technical training and are laborious difficult to use at the point of care. ICT is not only highly sensitive and specific but also rapid and cost-efficient, requiring minimal training of operators, making it attractive for field applications [37]. Here, we developed an ICT using purified rTgROP14, recombinant protein A and mAb TgROP14-5D5 for serological detection of *T. gondii* in swine populations.

Among 436 porcine sera collected from farm pigs in Zhejiang, China, the new ICT identified 134 *T. gondii*-positive sera (30.7%). The seroprevalence obtained in the present study was lower than that from Chongqing (66.5%), Guizhou (42.0%) and Xinjiang (35.8%), China, and higher than that from Heilongjiang (10.3%), Qinghai (11.8%) and Guangdong (12.0%), China [38,39]. The prevalence of *T. gondii* infection in pigs varies across districts in China. Moreover, the overall seroprevalence found in our study was lower than the 53.4% of 813 pigs examined by a commercial ELISA kit in Zhejiang, China, between 2009 and 2010 [40]. The decrease in seroprevalence may be due to improved feeding conditions and enhanced management. Nevertheless, the relatively high seropositivity level found in this study suggests an endemic circulation of *T. gondii* in this region.

Several ICTs have been developed for detection *T. gondii* infections to date. An ICT based on recombinant TgSAG2 was established for detection of anti-*T. gondii* antibodies in cats with relative sensitivity and specificity of TgICT of 100 and 94.5%, respectively, compared with LAT and 97.2 and 95.8% compared with ELISA [16]. An additional ICT based on GRA7 was developed for detection of *T. gondii* infection in swine populations. The relative sensitivity and specificity were 80% and 100% when iELISA was used as a reference [18]. Previously, the sensitivity and specificity of a few other approaches were computed as 45.9 and 96.9%, respectively, for LAT; 82.9 and 90.29% for MAT; 29.4 and 98.3% for IHAT; and 72.9 and 85.9% for ELISA [41]. Compared with the above methods, the ICT developed in this study exhibited higher sensitivity but lower relative specificity. In general, the novel ICT based on rTgROP14 and TgROP14-5D5 mAb is a promising candidate as a practical serological diagnostic test for the clinical investigation of *T. gondii* infection.

## 5. Conclusions

In this study, we showed that the rhoptry protein TgROP14 can recognize positive serum of *T. gondii* but not negative serum. The mAb TgROP14-5D5 can specifically recognize *T. gondii* antigens. The ICT using purified rTgROP14, recombinant protein A and mAb TgROP14-5D5 achieved good specificity and sensitivity. The novel ICT has potential for serological detection of *T. gondii* at the point of care in swine populations.

## Figures and Tables

**Table 1:** Immunological characteristics of TgROP14-5D5 mAb and TgROP14-1E9 mAb.

mAb	Isotype	Titer	Additivity Index
5D5	IgG2a	1:3.3 × 10^7^	22.80%
1E9	IgG3	-	

**Table 2:** Comparing the number of positive and negative samples detected by the ICT and IHA methods.

ICT	IHA (Reference Standard)		Total
	No. of Positive	No. of Negative	
No. of positive	90	44	134
No. of negative	9	293	302
Total	99	337	436

**Table 3:** Comparing the percentage of positive and negative samples detected by the ICT and IHA methods.

Test	No. (%) of Positive Samples	No. (%) of Negative Samples
ICT	134 (38.7)	302 (87.3)
IHA	99 (22.7)	337 (77.3)

**Table 4:** Sensitivity, specificity, PPV and NPV of *T. gondii* diagnostic tests using a commercially available IHA and a composite reference standard as comparators.

Test	% (95% Confidence Interval)			
	Sensitivity	Specificity	PPV	NPV
IHA as reference				
ICT	90.9 (83.6–95.1)	86.9 (82.9–90.1)	67.2 (57.4–75.6)	97.0 (94.6–98.4)
Composite reference standard				
ICT	93.7 (88.5–96.7)	100 (98.7–100)	100 (97.4–100)	97.0 (94.4–98.4)

## Data Availability

Data is contained within the article.

## Supplementary Materials

The following supporting information can be downloaded at: https://www.mdpi.com/article/10.3390/ani12151929/s1, Figure S1. Transmembrane domain prediction of TgROP14 protein by TMHMM Server v.2.0; Figure S2. Uncropped blots from Figure 2; Figure S3. Uncropped blots from Figure 3; Figure S4. Additional specificity of the ICT strip using sera of pigs infected with different common viruses as specified. From left to right: negative control, *Toxoplasma gondii* (Tg), porcine reproductive and respiratory syndrome virus (PRRSV), swine type-O foot-and-mouth disease virus (FMDV O-type), swine type-A foot-and-mouth disease virus (FMDV A-type) and swine fever virus (SFV); Table S1. Densitometry readings of each band for Western blot figures.
