# Supplementary figures and images for: Development of an Immunochromatographic Test Based on Rhoptry Protein 14 for Serological Detection of Toxoplasma gondii Infection in Swine
Source: Animals (Basel). 2022 Jul 28;12(15):1929. doi: 10.3390/ani12151929 (PMC9367252; doi:10.3390/ani12151929)

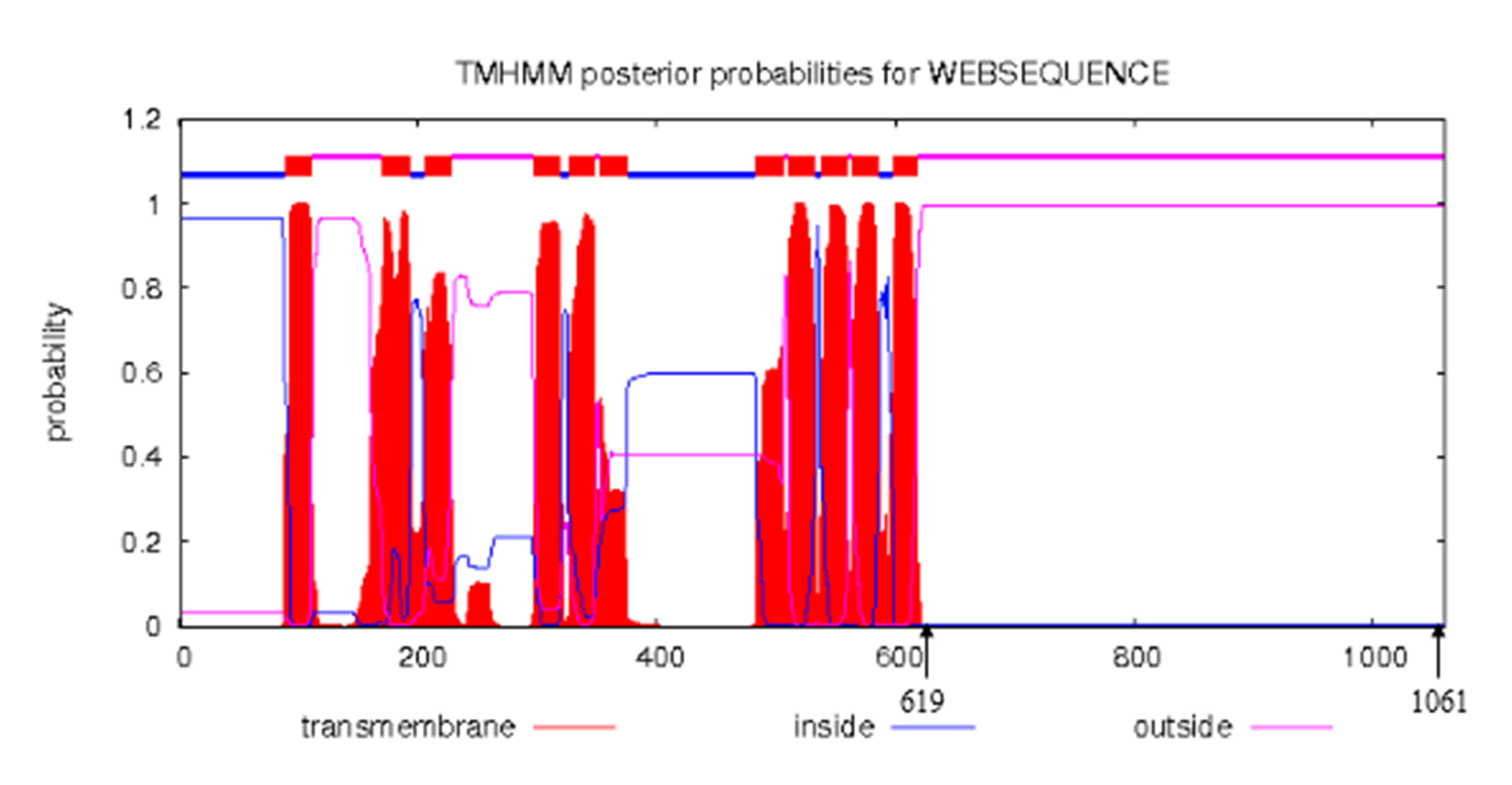

Supplement: Supplementary file 1 [file animals-12-01929-s001.zip › Supplementary Files/Figure S1.tif]

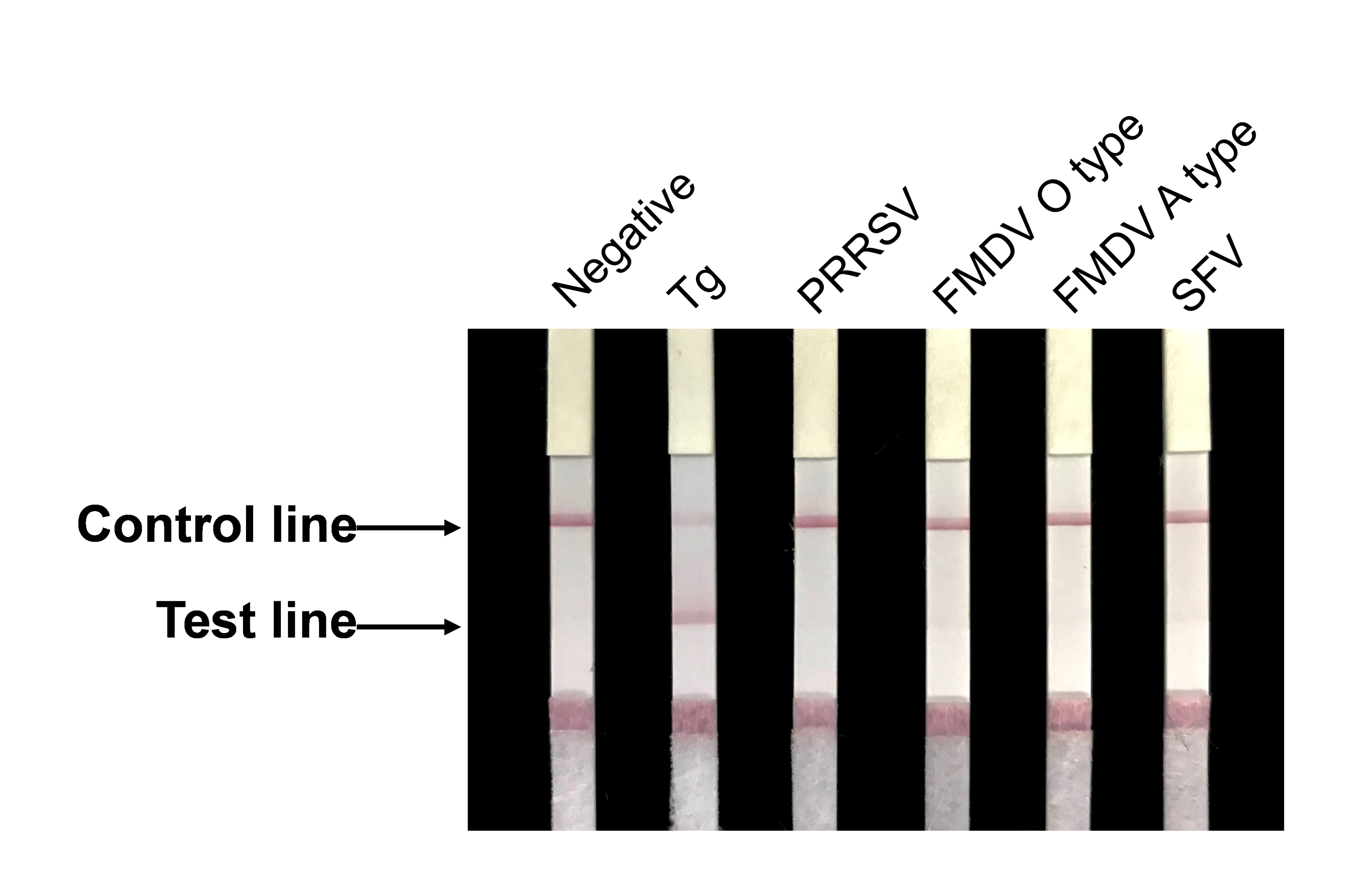

Supplement: Supplementary file 1 [file animals-12-01929-s001.zip › Supplementary Files/Figure S4.tif]
